# Awareness and Interest in Uterus Transplantation over Time: Analysis of Those Seeking Surgical Correction for Uterine-Factor Infertility in the US

**DOI:** 10.3390/jcm12134201

**Published:** 2023-06-21

**Authors:** Liza Johannesson, Giuliano Testa, Menas M. Beshara, Briget da Graca, Jessica R. Walter, Cristiano Quintini, Nawar Latif, Koji Hashimoto, Elliott G. Richards, Kathleen O’Neill

**Affiliations:** 1 Annette C. and Harold C. Simmons Transplant Institute, Baylor University Medical Center, Dallas, TX 75246, USA; 2 Department of Obstetrics and Gynecology, Baylor University Medical Center, Dallas, TX 75246, USA; 3 TX A&M College of Medicine, Texas A&M University, Dallas, TX 75231, USA; 4 Department of Obstetrics & Gynecology, Northwestern University, Chicago, IL 60611, USA; 5 Department of General Surgery, Digestive Disease Institute, Cleveland Clinic Abu Dhabi, Abu Dhabi, United Arab Emirates; 6 Division of Gynecologic Oncology, Department of Obstetrics and Gynecology, University of Pennsylvania, Philadelphia, PA 19107, USA; 7 Department of General Surgery, Digestive Disease & Surgery Institute, Cleveland Clinic, Cleveland, OH 44103, USA; 8 Obstetrics and Gynecology and Women’s Health Institute, Cleveland Clinic, Cleveland, OH 44103, USA; 9 Division of Reproductive Endocrinology and Infertility, Department of Obstetrics and Gynecology, University of Pennsylvania, Philadelphia, PA 19107, USA

**Keywords:** uterus transplantation, recipient, living donor, hysterectomy, infertility, parity

## Abstract

This study describes the characteristics of women who contacted an active program performing uterus transplantation (UTx) in the US, expressing interest in becoming a uterus transplant recipient or a living donor. Basic demographic and self-reported clinical information was collected from women who contacted any of the three US UTx programs from 2015 to July 2022. The three centers received 5194 inquiries about becoming a UTx recipient during the study timeframe. Among those reporting a cause of infertility, almost all of the reports (4066/4331, 94%) were absence of a uterus, either congenitally (794/4066, 20%) or secondary to hysterectomy (3272/4066, 80%). The mean age was 34 years, and 49% (2545/5194) had at least one child at the time of application. The two centers using living donors received 2217 inquiries about becoming living donors. The mean age was 34 years, and 60% (1330/2217) had given birth to ≥1 child. While most of the UTx clinical trial evidence has focused on young women with congenital absence of the uterus, these results show interest from a much broader patient population in terms of age, cause of infertility, and parity. These results raise questions about whether and to what extent the indications and eligibility criteria for UTx should be expanded as the procedure transitions from the experimental phase to being offered as a clinical treatment.

## 1. Introduction

Uterus transplantation (UTx) is an effective and curative treatment for women with uterine-factor infertility (UFI) [1]. In the United States, the first (although ultimately unsuccessful) attempt at UTx occurred in 2016, and the first successful live birth occurred in 2017 [2]. These events drew considerable media attention, and since then, interest in the procedure has progressively increased in the number of media articles, peer-reviewed publications, and interested applicants (Supplementary Figure S1). While UTx has expanded to more centers, the surgical volume has been outpaced by the number of applicants. From February 2016 to July 2022, 37 UTx were performed in the US [1], corresponding to <1% of women applying to US UTx programs.

In the US to date, the most common indication for completed UTx is the congenital absence of the uterus (Mayer-Rokitansky-Küster-Hauser, or MRKH, syndrome) [1]. This is also true internationally; the first publication from the Registry of the International Society of Uterus Transplantation, with participating centers from Europe, China, the Middle East, and Latin America, reported that 44 of the 45 recipients had congenital MRKH [3].

Currently, no published reports have aggregated data of the characteristics of interested individuals contacting UTx transplant programs. Several single centers have reported that most individuals contacting their center had causes of UFI other than MRKH, including hysterectomy for malignancy, benign conditions, or obstetric complications [4,5,6,7,8]. In two US centers, almost two-thirds of women inquiring about UTx had UFI secondary to hysterectomy, including large proportions who had undergone hysterectomy for benign conditions at a young age [4,7].

As UTx transitions from experimental to clinical practice [9], it is important to assess how well the population ultimately interested in undergoing the procedure matches the population for which safety and effectiveness data have been established. Both technical and ethical considerations for UTx may differ by recipient characteristics, such as the cause of infertility and parity [6]. Knowing who, beyond women with MRKH, is aware of and interested in undergoing UTx will help determine the extent to which the current evidence can support the expanded offering of the procedure, as well as where additional clinical trials are needed.

Similar considerations apply to women interested in becoming living uterus donors. The majority of uterus donors in the US have been living and nondirected [1], motivated by helping others experience pregnancy and contributing to science [10]. According to the Organ Procurement and Transplantation Network (OPTN) in the US, living, nondirected donation is an ethically justifiable form of organ donation provided that a strict standard of informed consent is followed, the competent potential donor undergoes appropriate evaluation, and organs are allocated in an equitable manner (ref: Ethics—Living Non-Directed Organ Donation—OPTN (hrsa.gov)). Data from other countries, in contrast, indicate a predominance of living directed donors, particularly mothers or sisters of recipients [3]. While limited characteristics (e.g., age, body mass index, comorbidities, directed vs. nondirected donation) of living donors have been included in the multicenter reports of UTx transplantation [1,3], information about the broader population of women interested in donation has been reported by only one center [7]. Understanding those interested in becoming a living donor (directed or nondirected) is important to assess the prevalence of donors meeting clinical criteria to maximize successful UTx outcomes and for whom donation carries acceptable risks, particularly if the use of UTx expands more broadly in the full population of women with UFI [11,12].

In this study, we describe the characteristics of women showing interest in becoming a UTx recipient or living donor who contacted an active program performing UTx in the US. These data may provide important insights into which individuals pursue this relatively new procedure and whether their numbers have been underestimated.

## 2. Materials and Methods

Data were derived from the following three US institutions: Baylor University Medical Center (BUMC), Dallas; Cleveland Clinic Foundation (CCF), Cleveland; the University of Pennsylvania (UPenn), Philadelphia. This study was approved by the local institutional review board at all three participating institutions. During the study period, CCF allowed for deceased uterus donation only, whereas BUMC and UPenn permitted both living and deceased uterus donation. Potential recipients and donors contacted the UTx clinical program in a myriad of ways including by email, phone, institutional trial listing platform, and an institutional UTx-specific website. Basic demographic and self-reported medical information were collected and managed using the Research Electronic Data Capture (REDCap™) platform, hosted at each of the institutions. REDCap is a secure, web-based platform designed for research data capture [13,14]. The applicants described in this study independently contacted the institutions, with no advertising or recruiting being performed. The registration of interested applicants started in 2015 (recipients, CCF), 2016 (recipients and living donors, BUMC), 2017 (recipients, Upenn), and 2019 (living donors, Upenn). BUMC and CCF did not register applications during 2020 due to the COVID-19 pandemic.

We report demographic data and descriptive characteristics of women expressing interest in becoming either a uterus recipient or living donor. We further compared demographic data for participants living in states with and without infertility insurance mandates and states with and without case laws prohibiting gestational surrogacy. Pearson chi-squared tests were used to compare distributions of categorical variables and two-sample *t* tests were used to compare continuous variables.

## 3. Results

### 3.1. Applicant Characteristics: Potential Recipients

**Figure 1:**
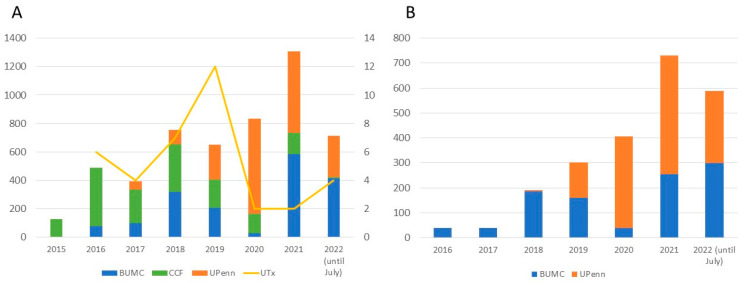
Recipient (**A**) and living donor (**B**) interest for uterus donation and transplantation at Baylor University Medical Center (BUMC), Dallas; Cleveland Clinic Foundation (CCF), Cleveland; the University of Pennsylvania (UPenn), Philadelphia, 2015 to July 2022. Note that BUMC did not record interest during 2020 (COVID pandemic).

**Figure 2:**
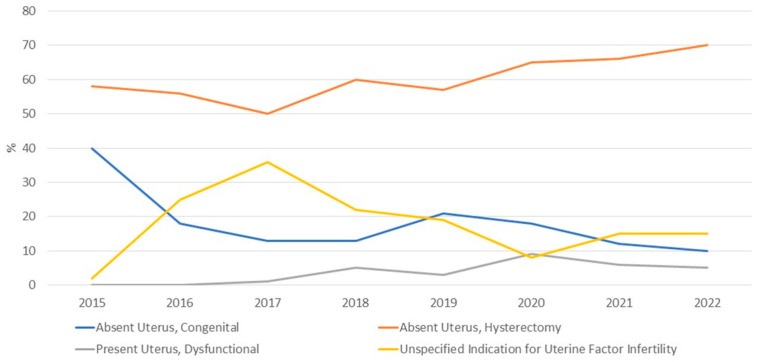
Indication for uterus transplant per year for potential recipients expressing interest in uterus transplantation at Baylor University Medical Center, Dallas; Cleveland Clinic Foundation, Cleveland; the University of Pennsylvania, Philadelphia, 2015 to July 2022.

**Figure 3:**
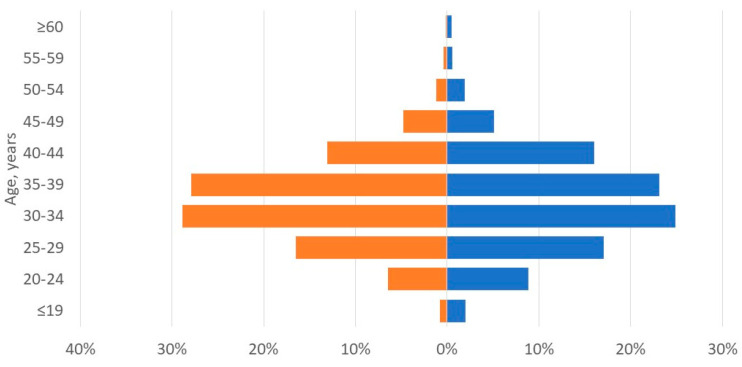
Age of potential uterus recipients (orange) and living donors (blue) expressing interest in uterus donation and transplantation at Baylor University Medical Center, Dallas; Cleveland Clinic Foundation, Cleveland; the University of Pennsylvania, Philadelphia, 2015 to July 2022.

**Figure 4:**
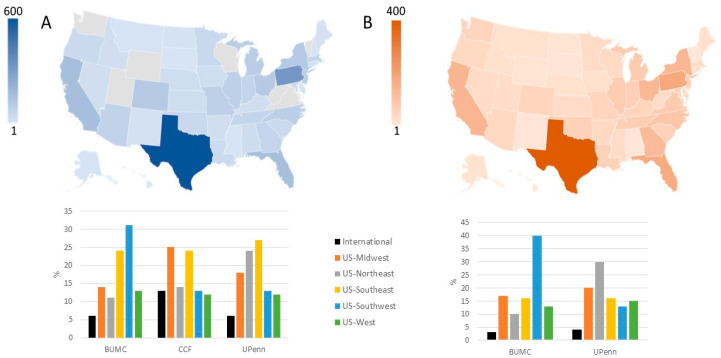
Geographical origin of the recipients (**A**) and living donors (**B**) expressing interest in uterus transplantation and donation at Baylor University Medical Center (BUMC), Dallas; Cleveland Clinic Foundation (CCF), Cleveland; the University of Pennsylvania (UPenn), Philadelphia, 2015 to July 2022. Site-specific application origin is shown below the maps.

A total of 5194 women applied to receive UTx from US-based clinical trials between 2015 and July 2022 (Figure 1A). Recipient demographics are shown in Table 1. Among those reporting an indication for UTx (4331/5194, 83%), the majority did not have a uterus (4066/4331, 94%), while a significantly smaller proportion had a present but dysfunctional uterus (265/4331, 6%) (Table 2). Among those without a uterus, 80% (3272/4066) had undergone a previous hysterectomy, and 20% (794/4066) had congenital uterine absence. The yearly changes of indication for UTx are shown in Figure 2. The mean (standard deviation [SD]) age at the time of UTx application was 34 (7) years (Figure 3), and the mean (SD) body mass index was 29 (8) kg/m^2^. Of the potential recipients, 49% (2545/5194) had at least one child at the time of the application, and 11% (571/5194) had four or more children. Most potential recipients reporting their geographical origin (83% [4311/5194]) came from within the United States (93% [3999/4311]), and a smaller portion was international (7% [312/4311]) (Figure 4A). Among the applicants from the United States, 27% (1079/3999) were from the Southeast, 21% (840/3999) from the Southwest, 19% (760/3999) from the Midwest, 19% (760/3999) from the Northeast, and 14% (560/3999) from the West. Overall, 19% of applicants lived in states with active UTx clinical trials (TX, Ohio, and Pennsylvania). Approximately two-thirds of potential recipients applied from the 29 states without infertility insurance mandates (61%), and 4% applied from states with statutes or published case law prohibiting gestational surrogacy (Louisiana, Michigan, and Nebraska). There were no clinically significant differences in the demographics of recipient applicants applying from states with active UTx programs, favorable insurance mandates, or restrictive surrogacy laws.

### 3.2. Applicant Characteristics: Potential Donors

A total of 2217 women applied to become a living uterus donor for UTx between 2015 and July 2022 (Figure 1B). Donor demographics are shown in Table 1. The mean (SD) living donor age at the time of uterus donation application was 34 (8) years (Figure 3), and the mean (SD) body mass index was 28 (7) kg/m^2^.

Most potential living donors had previous children (60%, 1330/2217), with the majority having given birth to two or three children (37%, 820/2217); 39% (865/2217) of the living donor applicants were nulliparous.

Most potential living donors who reported geographical origin (97%, 2173/2217) came from within the United States (96%, 2128/2173), and a smaller portion were international (4%, 45/2173) (Figure 4B). Among the applicants from the United States, 26% (553/2128) were from the Southwest, 22% (468/2128) from the Northeast, 20% (426/2128) from the Midwest, 17% (362/2128) from the Southeast, and 15% (319/2128) from the West.

## 4. Discussion

This paper provides, for the first time, aggregate national data describing the trends and characteristics of individuals expressing interest in active centers in becoming UTx recipients or living donors during the first 5 years of UTx in the US. Interest has increased over time, with a noticeable rise following the December 2017 media coverage of the first live birth after UTx in the United States [15,16]. In addition, interest from potential recipients and donors continued to rise even with the nationwide halt in performing UTx in 2020 due to the COVID-19 pandemic. While most of the observed interest was from domestic applicants, 7% of the individuals interested in becoming recipients and 4% of those interested in becoming living donors were international. The predominant indication for UTx among individuals interested in becoming recipients was the absence of the uterus due to hysterectomy. The number increased over time, suggesting that interest in pursuing UTx extends far beyond the estimated 15,000 women of reproductive age with MRKH living in the US.

The number of women interested in becoming recipients was more than double the number interested in becoming donors. While some of the shortfalls may be due to the use of deceased rather than living donors in one program [4], and some women interested in becoming recipients will likely be able to find directed donors among their family and friends (as has been the strategy employed in some UTx clinical trials [17]), this data point provides further support for the argument that an allocation system from nondirected living donors and deceased donors will be needed to ensure equitable access for US women interested in UTx. It is also notable that nearly a third of interested donors were nulliparous. Though UTx has been performed using nulliparous deceased donors, the acceptability, eligibility, and ethics of utilizing nondirected nulliparous living donors remains controversial, given not insignificant surgical risks associated with uterus donation and sterilization resulting from donation [18].

The demographic characteristics of women expressing interest in becoming recipients observed here are largely consistent with smaller preliminary reports [4,7], although the mean age in our data was higher (34 years vs. 28 and 32 years), as was the proportion who already had at least one child (49% vs. 17% and 47%). These observations are likely related to the rising proportion of women with uterine absence secondary to hysterectomy, as previous reports have found this subgroup to have a higher average age than those with congenital uterine absence [4] and to be more likely to have a biological child.

The importance of the observed increase in applicants with UFI secondary to hysterectomy cannot be overstated. It suggests a profound underestimation of clinical need and a current mismatch between the most prevalent patient population interested in UTx and the cohort actually completing transplantation. This is true not only in the US but worldwide, where congenital absence to date is by far the most common indication for UTx [1,3]. Existing and emerging UTx programs may need to consider revising inclusion criteria (e.g., recipient age and parity) to better align eligibility criteria for patients with an antecedent hysterectomy. Other revisions—or additional clinical trial evidence generation in a cohort better reflecting the interested patient population—may be needed to address the differing considerations for UTx according to the cause of UFI [6]. These considerations impact not only selection criteria but also the risks and benefits weighed in the ethical evaluation of UTx, which, to date, has focused largely on recipients who have not had the opportunity to experience pregnancy and/or have personal or legal contraindications to surrogacy and adoption [19,20,21,22,23,24]. For example, patients with a prior peripartum hysterectomy may have immunological issues related to anti-HLA response (due to pregnancy and/or prior transfusions received during the obstetric complications leading to hysterectomy), adding to the medical risks to the recipient that must be considered [6]. Additionally, in the case of women who have previously carried their own pregnancies and are raising the resulting children, the question arises as to whether the benefits of the opportunity to experience an additional pregnancy are sufficient to outweigh the medical risks to the recipient and potentially a living donor; this may differ from the evaluation of benefits vs. risks for women who have never previously had the opportunity to experience pregnancy. While women in both situations stand to benefit in terms of the opportunity to bond with their child during the experience of pregnancy, only the latter group also receives the benefits related to the female identity, such as feeling like a “complete” woman [25,26]. There is, further, the question of whether nondirected living donors—who have previously endorsed the opportunity to help other women experience pregnancy as one of the prime motivations to donate [10]—would still be willing to donate when nearly half of the recipients have already carried one or more children.

The increasing predominance of hysterectomy as the indication for UTx among interested US women contrasts with European UTx reports. While 63% overall (and 70% in 2022) of US UTx recipient inquiries were from women with a prior hysterectomy, the same was true for only ~25% of the inquiries reported by UTx programs in France and Germany [6,8]. The differences may be primarily driven by hysterectomy for benign conditions, which represented a minority (25%) of the hysterectomies preceding UTx inquiries to the French program [6] but the majority (50–73%) at US centers [4,7]. As has been previously noted, these findings warrant a reexamination of the “culture of hysterectomy in the United States that results in permanent sterility when performed in a reproductive-aged population” [4]. Our finding that 57% of the inquiries to the three US UTx programs about becoming UTx recipients were by women reporting a hysterectomy for benign conditions underscores this concern.

The predominant attributes of these prospective recipients are at odds with the hypothetical patients for whom surveys of physicians indicate they would regard UTx as an appropriate treatment option. In a 2018 survey of physicians at the Mayo Clinic, 45% agreed UTx should be considered for a 22-year-old with congenital absence of the uterus, but only 19% thought the same for a 32-year-old woman who already has children and lost her uterus to hysterectomy [27], yet the latter hypothetical recipient is most reflective of the majority of those contacting US UTx transplant programs. The 2018 Mayo survey, like others, suggests that physician reluctance to consider UTx as a treatment option for women with UFI is predominantly secondary to concerns about complications for the recipient [28]. It is important to note that this survey was conducted before much of the safety and efficacy evidence from UTx was available. Updated surveys are needed to evaluate current attitudes towards UTx as a treatment option and should investigate whether or to what extent attitudes might differ according to clinical indications, recipient characteristics, and donors and how those compare to the patient populations expressing interest in this procedure.

With respect to living donor inquiries, the only available comparison is an early report by the BUMC program [7]. The expanded data shared here demonstrate a different profile of interested candidates, with an average age of 34 years (vs. 40 years previously reported [7]) and only 60% (vs. 90% [7]) having already had children. Additional data are needed to determine the extent to which women interested in becoming living donors meet the eligibility criteria required by uTx programs. The current data do show a relatively good match to the eligible age range of from 30 to 50 years listed by both BUMC [29] and uPenn [30], with 69% meeting this criterion, but detailed medical history information would be needed to evaluate the other criteria.

Some limitations must be considered when interpreting the results of this study. First, it was a retrospective observational study using the administrative data available from UTx programs that had been contacted by women interested in becoming recipients or living donors. As such, it is limited to considering the variables for which data were available rather than all that might be of interest, as a prospective study would facilitate. Second, while our results reflect the characteristics of the women who have contacted the US UTx programs about becoming recipients or donors, they may not be generalizable to all women interested in these options as follows: characteristics may differ between the early adopters who were actively seeking information early in the clinical trial phase of UTx and later adopters. Additionally, although many individuals applied to the programs, the volume and characteristics of interested recipients may change outside the context of clinical trials. Furthermore, information on race and ethnicity was not routinely collected in the forms submitted by interested parties. Most US UTx recipients have been white and non-Hispanic. This may be partly due to these procedures having been performed in the context of clinical trials, for which the barriers to participation for racial/ethnic minorities, particularly related to historical fear and mistrust, are well documented [31,32], as well as the requirement to cover costs of the in vitro fertilization steps in the process. Understanding how race and ethnicity affect interest in accessing this treatment for UFI will be important moving forward. Other limitations include the incomplete data from 2020 when centers stopped performing UTx due to the COVID-19 pandemic and did not record information regarding interested potential candidates. In addition, some individuals might have contacted multiple programs. As the data available do not enable the identification of such duplicates, it is possible that the aggregate data presented here overestimate the interest in becoming a UTx recipient or living donor.

## 5. Conclusions

In conclusion, the results reported here show that increasing numbers of women have contacted US UTx programs over time, and the women interested in becoming recipients differ substantially from the assumed target population of young women with congenital absence of the uterus and no previous pregnancies. We observed substantial interest from women in their mid to late 30s and 40s, from women who lacked a uterus due to hysterectomy, and from women who already had one or more children. The improved understanding this study provides of the interested patient population is important as UTx becomes more widely offered outside the context of clinical trials. Findings call for consideration of, and possible updates to, patient selection criteria that reflect the population interested in UTx, as well as the safety and effectiveness data available from the clinical trials and the ethical evaluation of UTx, which has assumed that a substantial benefit to the recipient was the opportunity to experience a first pregnancy, rather than an additional one. These factors will similarly be important to consider as allocation strategies for deceased and nondirected living donor uteri are developed. Optimizing recipient and donor criteria to ensure successful outcomes and equitable access to those outcomes are key challenges for UTx in its next phase of evolution, and understanding who is interested in becoming a recipient or donor is critical to meeting those challenges. Further research will be needed to fully describe those interested populations, but the results presented here provide important insights that can guide the framing of that work.

## Figures and Tables

**Table 1:** Characteristics of applicants for uterus transplantation and living donation at Baylor University Medical Center, Dallas; Cleveland Clinic Foundation, Cleveland; the University of Pennsylvania, Philadelphia, 2015–July 2022.

Variable	Recipients	Living Donors
Candidates expressing interest, n	5194	2217
Age, years, mean ± SD	34 ± 7	34 ± 8
Body mass index, kg/m^2^, mean ± SD	29 ± 8	28 ± 7
Previous children	2545 (49%)	1330 (60%)
None	1039 (20%)	865 (39%)
1	571 (11%)	244 (11%)
2	727 (14%)	510 (23%)
3	676 (13%)	310 (14%)
≥4	571 (11%)	266 (12%)
Not reported	1610 (31%)	22 (1%)
Geographic origin		
United States	3999 (77%)	2128 (96%)
International	312 (6%)	45 (2%)
Not reported	883 (17%)	44 (2%)
Indication for uterus transplantation		
Absent uterus, congenital	796 (15%)	NA
Absent uterus, hysterectomy	3259 (63%)	NA
Present uterus, dysfunctional	251 (5%)	NA
Not reported	888 (17%)	NA

NA indicates not applicable.

**Table 2:** Reported indication for uterine factor infertility among potential recipients for uterus transplantation at Baylor University Medical Center, Dallas; Cleveland Clinic Foundation, Cleveland; the University of Pennsylvania, Philadelphia, 2015–July 2022.

Category	Indication	N
Absent Uterus, Congenital		796
	Mullerian Anomaly (Includes MRKH)	749
	Swyer Syndrome	1
	Transgender Female	41
	Turners Syndrome	1
	CAIS	3
	Intersex, no Female Sex Organs	1
Absent Uterus, Hysterectomy		3259
Gynecological Indication		
Abnormal bleeding	Abnormal bleeding, Unspecified	58
	Menorrhagia	65
	Endometrial Ablation	1
Contraception	Contraception	8
	Complications of contraception *	21
Infection	Pelvic inflammatory disease	5
	Infection, Unspecified	16
Malformations	Bicornuate Uterus	2
	Hypoplasia	5
	Outflow Obstruction/Hematometra	3
	Septate Uterus	1
	Malformation, Unspecified	8
Malignancy or Premalignancy	Cervical dysplasia	64
	Malignancy, Adenosarcoma	2
	Malignancy, Appendix	2
	Malignancy, Breast	1
	Malignancy, Cervical	120
	Malignancy, Colon	2
	Malignancy, Endometrial	56
	Malignancy, Gestational Trophoblastic Disease	6
	Malignancy, Ovarian	17
	Malignancy, Placental	3
	Malignancy, Rhabdomyosarcoma	2
	Malignancy, Unspecified	48
Pain	Congested Pelvic Syndrome	9
	Chronic Pain	29
	Dysmenorrhea	15
	Endometriosis	298
Structural Abnormalities	Adenomyosis	57
	Asherman syndrome	8
	Myomas	320
Trauma	Trauma, Sexual	5
	Trauma, Unspecified	29
Urogynecological indication	Fistula	1
	Prolapse	55
	Reconstruction surgery	1
Other	Complication of Surgery	6
	Cysts	23
	Scarring, Unspecified (Includes Complication of Radiation)	11
	PCOS	10
Obstetrical Indication	Abnormal Placentation	66
	Complication of Miscarriage/Abortion	16
	Complication of Pregnancy, Unspecified	22
	Complications of Ectopic Pregnancy	6
	Complications of Molar Pregnancy	3
	Postpartum Hemorrhage	289
	Postpartum Infection	9
	Uterine Rupture at Delivery	28
Other	Claim of Malpractice	33
	Family Pressure	9
	Personal Decision	3
Unspecified Indication for Hysterectomy		1382
Present Uterus, Dysfunctional		251
	Adenomyosis	3
	Asherman Syndrome	6
	Endometrial Ablation	19
	Endometriosis	11
	Hypoplasia	3
	Malignancy, Unspecified	4
	Myomas	14
	Trauma, Unspecified	4
	Present but Dysfunctional Uterus, Unspecified	187
Unspecified Indication for Uterine Factor Infertility		888
Total		5194

MRKH indicates Mayer-Rokitansky-Küster-Hauser; * Includes tubal ligation, IUD, and Essure.

## Data Availability

The data presented in this study are available on request from the corresponding author.

## Supplementary Materials

The following supporting information can be downloaded at: https://www.mdpi.com/article/10.3390/jcm12134201/s1, Figure S1: (A) Increasing US interest in uterus transplantation as evidenced by Google Trends internet search data. Interest in uterus transplantation peaked in 2016 with the first attempted uterus transplantation in the United States by Cleveland Clinic and again in late 2017/early 2018 with the first successful uterus transplantation in the United States by Baylor Scott and White. Per Google Trends (https://trends.google.com/), “numbers represent search interest relative to the highest point on the chart for the given region and time. A value of 100 is the peak popularity for the term. A value of 50 means that the term is half as popular. A score of 0 means there was not enough data for this term”. Using this metric and comparing the average from 2010 (0.03 ± 0.03) to 2022 (0.18 ± 0.07), interest in uterus transplantation has risen by 15.7%. (B) Relative percentage of search queries by subregion showing that the number of queries for uterus transplantation (relative to other search queries for that region) was highest in states with active programs.
