# Supplementary material for: Awareness and Interest in Uterus Transplantation over Time: Analysis of Those Seeking Surgical Correction for Uterine-Factor Infertility in the US
Source: J Clin Med. 2023 Jun 21;12(13):4201. doi: 10.3390/jcm12134201 (PMC10342774; doi:10.3390/jcm12134201)
Supplement: Supplementary file 1 [file jcm-12-04201-s001.zip › jcm-2418370-supplementary.pdf]

Journal of
Clinical Medicine

MDPI

Supplementary Material

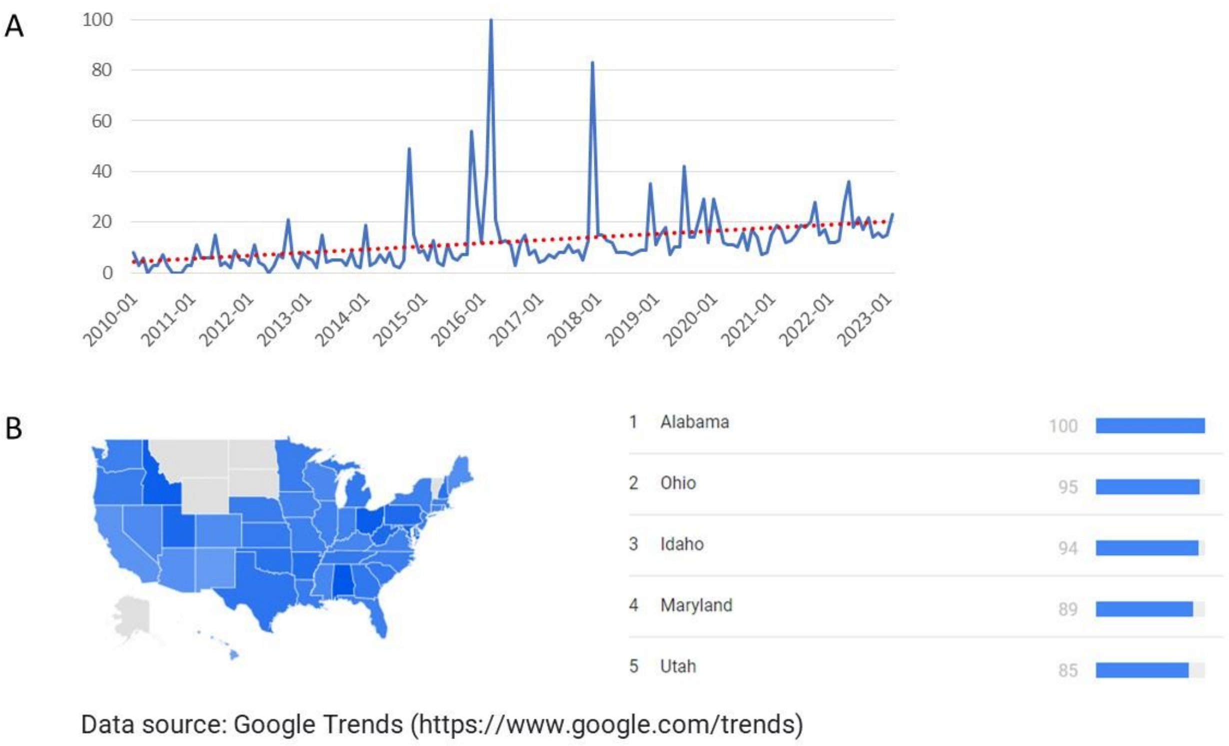

Supplementary Figure S1. (A) Increasing US interest in uterus transplantation as evidenced by Google Trends internet search data. Interest in uterus transplantation peaked in 2016 with the first attempted uterus transplantation in the United States by Cleveland Clinic and again in late 2017/early 2018 with the first successful uterus transplantation in the United States by Baylor Scott and White. Per Google Trends (https://trends.google.com/), "numbers represent search interest relative to the highest point on the chart for the given region and time. A value of 100 is the peak popularity for the term. A value of 50 means that the term is half as popular. A score of 0 means there was not enough data for this term". Using this metric and comparing the average from 2010 (0.03 ± 0.03) to 2022 (0.18 ± 0.07), interest in uterus transplantation has risen by 15.7%. **(B)** Relative percentage of search queries by subregion showing that the number of queries for uterus transplantation (relative to other search queries for that region) was highest in states with active programs.
